# Supplementary material for: Social Stimulation by the Owner Increases Dogs’ (Canis familiaris) Social Susceptibility in a Food Choice Task—The Possible Effect of Endogenous Oxytocin Release
Source: Animals (Basel). 2022 Jan 25;12(3):296. doi: 10.3390/ani12030296 (PMC8833464; doi:10.3390/ani12030296)
Supplement: Supplementary file 1 [file animals-12-00296-s001.zip › animals-1531668-supplementary.pdf]

Experiment I

Table S1. Summary of subjects for the four experimental groups. "Addr.": addressing demonstration, "N-Addr.": non-addressing demonstration.

Pre-treatment:	Stimulating		Ignoring		Statistics	*p*
Demonstration context:	Addr.	N-Addr.	Addr.	N-Addr.		
Nr. of subjects	19	22	17	24	-	-
Age in months (mean ±SE)	47.9 ± 6.9	56.8 ± 8.8	42.6 ± 6.8	47.8 ± 7.9	ANOVA $F_{(3,81)} = 0.55$	0.65
Nr. of males & females	12 & 7	12 & 10	7 & 10	13 & 11	chi^2 test $\chi^2_{(3)} = 1.76$	0.62
Dogs with training certificate & untrained dogs	13 & 6	16 & 6	12 & 5	16 & 8	chi^2 test $\chi^2_{(3)} = 0.22$	0.97

Experiment II

Table S2. Summary of subjects for the two experimental groups.

Pre-treatment:	Oxytocin	Placebo	Statistics	*p*
Nr. of subjects	15	17	-	-
Age in months (mean ±SE)	56.8 ± 4.9	53.1 ± 10.7	indep. t-test $t_{(30)} = 0.295$	0.77
Nr. of males & females	8 & 7	10 & 7	Fisher's exact test	1.00
Dogs with training certificate & untrained dogs	12 & 3	11 & 6	Fisher's exact test	0.44
Time elapsed between Exp. I & II (in months, mean ±SE)	6.22 ± 0.74	7.07 ± 0.58	indep. t-test $t_{(30)} = 0.886$	0.38
Nr. of subjects that received Stim. & Ign. pre-treatment in Exp. I	6 & 9	8 & 9	Fisher's exact test	0.73
Change-in-bias score (mean ±SE) in Exp. I	1.47 ± 0.3	1.41 ± 0.56	indep. t-test $t_{(30)} = 0.083$	0.94
